# Effect of Different Activated Carbon as Carrier on the Photocatalytic Activity of Ag-N-ZnO Photocatalyst for Methyl Orange Degradation under Visible Light Irradiation

**DOI:** 10.3390/nano7090258

**Published:** 2017-09-05

**Authors:** Xiaoqing Chen, Zhansheng Wu, Zhenzhen Gao, Bang-Ce Ye

**Affiliations:** School of Chemistry and Chemical Engineering/The Key Laboratory for Green Processing of Chemical Engineering of Xinjiang Bingtuan, Shihezi University, Shihezi 832003, China; chenxq0405@126.com (X.C.); zhenzhengao1210@163.com (Z.G.); bcye@ecust.edu.cn (B.-C.Y.)

**Keywords:** photocatalytic degradation, zinc oxide, activated carbon, visible light, methyl orange

## Abstract

In order to enhance the photodegradation of methyl orange (MO) by ZnO under visible light irradiation, ZnO nanoparticles co-doped with Ag and N and supported on activated carbon (AC) with different properties were synthesized through the sol-gel method. The prepared photocatalysts were characterized in terms of the structure and properties through X-ray diffraction, N_2_ adsorption-desorption, ultraviolet-visible (UV-vis), diffuse reflectance spectroscopy, X-ray photoelectron spectroscopy, photoluminescence, and electron spin resonance. The photocatalytic activities of these photocatalysts followed the order: Ag-N-ZnO/ACs > Ag-N-ZnO > N, or Ag single-doped ZnO > commercial ZnO. This result was attributed to the small particle size, large surface area, narrow band gap, and high charge separation of Ag-N-ZnO/ACs. The Ag-N-ZnO/coconut husk activated carbon (Ag-N-ZnO/CHAC) exhibited the highest degradation efficiency of 98.82% for MO under visible light irradiation. This outcome was due to the abundant pore structure of Ag-N-ZnO/CHAC, resulting in stronger adsorption than that of other Ag-N-ZnO/ACs. Moreover, the degradation of MO on photocatalysis followed first order kinetics. The reactive species ·OH and ·O_2_^−^ played more important roles in the photocatalytic degradation of MO over composite photocatalyst. Ag-N-ZnO/CHAC photocatalyst exhibited higher photocatalytic activity than unsupported Ag-N-ZnO after five recycling runs.

## 1. Introduction

With the rapid development of global industry, more and more environmental problems—especially water pollution—are caused by organic pollutants. Numerous kinds of synthetic dyes pollutants which are widely applied in textile, food, and leather industries, have been discharged into the natural environment. And these pollutants are highly hazardous to the health of human beings [1]. Methyl orange (MO) is the most representative of all anionic azo dyes, which is not decomposed under ambient conditions and is usually resistant to classical biological treatment. Conventional physicochemical techniques, such as adsorption, coagulation, and reverse osmosis, are the most frequently developed to remove MO from aqueous solutions. However, these methods can merely accumulate MO and cannot transform the dye into harmless compounds [2,3,4,5,6]. Hence, the development of an efficient treatment method for MO is a scientific problem of considerable interest. Recently, numerous researchers have focused on photocatalytic degradation to handle dyes because this approach can completely mineralize dyes in wastewater.

Zinc oxide (ZnO) is one of the most popular photocatalysts due to its low toxicity, chemical stability, and superior photocatalytic properties [7,8,9]. ZnO presents a wide band gap of 3.37 eV, and can only be excited under UV light irradiation. However, sunlight consists of approximately 5% UV, 43% visible and 52% infrared [10] light, therefore the wide band gap of ZnO limits its photoactivity under visible light, which limits its applications that use sunlight as the energy source. Another drawback of ZnO is the rapid recombination rate of the photogenerated electron-hole pair, which limits the photodegradation reaction under normal conditions [11]. To overcome these drawbacks, numerous attempts have been made to prepare more efficient photocatalysts based on ZnO nanomaterials. One of the effective approaches to solve these shortcomings is to modify ZnO using metal and nonmetal dopants [12,13,14]. Numerous investigations on metal and nonmetal elements, such as Ag, Fe, N, and S, doped into ZnO have been conducted to enhance photocatalytic activity. Among these doped ZnO elements, ZnO single-doped with N can exhibit strong absorption in various reactions under visible light [15] and single Ag-doped ZnO can inhibit the recombination of photogenerated electrons and holes [12]. Therefore, it is worthwhile to investigate the effect of Ag and N co-doped-ZnO on the visible light response and inhibition of the recombination of photogenerated electrons and holes, which has considerable practical application value.

Moreover, it is difficult to recover the pure ZnO powder from the treated effluent, which limits the practical application of ZnO photocatalyst in industry. Finding effective approaches in order to solve the problem is very important. Until now, photocatalysts have been immobilized using various supports, which are generally porous materials. Many different porous materials have been investigated, such as activated carbon, zeolites, and glass [16,17,18]. Among these porous materials, AC is widely used as a photocatalyst carrier because of large specific surface area and rich functional groups [19,20,21]. Meanwhile, in the process of dye wastewater treatment, AC supported ZnO could be separated and reused easily from suspension, thus the lifetime of photocatalyst could be lengthened [22]. Furthermore, AC supported ZnO adsorbs a large number of pollutants and toxic intermediates formed after the initial degradation. Then, the photocatalytic degradation efficiency is improved [13]. However, the preparation of AC loaded, Ag and N co-doped-ZnO photocatalyst for the degradation of MO has yet to be reported. In addition, it is worth thoroughly researching the influence of ACs with different performance for supporting Ag and N co-doped ZnO photocatalyst on the photocatalytic degradation efficiency of MO.

The present work focuses on the synthesis of different AC loaded Ag-N-ZnO for MO removal. The crystal structure, morphology observation, and chemical properties of the photocatalysts were characterized by various techniques. The adsorption performance, the photocatalytic properties and stability of the photocatalysts were investigated using the removal rate of MO. The influences of different ACs prepared with coconut husk (CH), coal (C) and almond (A) (i.e., CHAC, coal activated carbon (CAC), and almond activated carbon (AAC)) to load Ag, N co-doped ZnO photocatalyst on photocatalytic degradation efficiency were also studied. The obtained results could be significant for the application of ZnO as a photocatalyst to remove MO.

## 2. Material and Methods

### 2.1. Materials

Zinc acetate was purchased from Tianjin Fuchen Chemical Reagent Co., Ltd. (Tianjin, China). Oxalic acid and urea were supplied by Tianjin Shengao Chemical Industry Limited Company (Tianjin, China). EtOH (anhydrous alcohol) was provided by Tianjin Fuyu Fine Chemical Co., Ltd. (Tianjin, China). MO and AgNO_3_ were obtained from Tianjin Yongsheng Fine Chemical Co., Ltd. (Tianjin, China). All reagents were of analytical grade and used without further purification.

### 2.2. Preparation Ag-N-ZnO/ACs

The photocatalysts were prepared based on previous studies [23]. In a typical synthesis, zinc acetate (2.196 g) was dissolved in absolute ethanol (EtOH) (60 mL) under stirring in water bath of 60 °C for 30 min and then it was named solution A. Solution B was prepared by mixing 5.040 g of oxalic acid dehydrate in 80 mL of EtOH under stirring in water bath of 50 °C for 30 min. Solution B was added dropwise to the warm solution A and the resulting solution was continuously stirred at room temperature for 1 h to get sol. The sol was aged in a sealed environment for 48 h until a homogenous gel was formed. Next, the product was dried in a vacuum oven at 80 °C for 24 h. Finally, ZnO was obtained by thermal treating at 400 °C for 2 h. To prepare Ag-N-ZnO, 0.034 g of AgNO_3_ and 0.9 g of urea was dissolved in solution A. Then, 2.0 g CHAC was dispersed in 200 mL ethanol for 1 h under sonication and 0.2 g ZnO was dispersed in 50 mL ethanol for 20 min. Both solutions were intermixed and the mixture was subsequently sonicated for 1 h and then stirred for 15 h at 300 rpm. The resulted samples were separated via centrifugation and dried at 80 °C and noted as Ag-N-ZnO/CHAC. Ag-N-ZnO/CAC and Ag-N-ZnO/AAC were prepared by the same method.

### 2.3. Characterization Methods

The X-ray diffraction (XRD) patterns of all samples were analyzed using a Rigaku Giegerflex D/Max B diffractometer (Rigaku Corporation, Tokyo, Japan) with Cu-Kα radiation. The scanning step and velocity used was 0.02° and 0.01°/minutes, respectively. The samples were scanned in the angle range of 10°–80°. The pore volume, pore size distribution, and specific surface area of samples were characterized by N_2_ adsorption at 77 K using a surface area analyzer (Micromeritics, ASAP 2020, Norcross, GA, USA). UV-Visible absorbance spectra to determine the optical band gap of the photocatalysts were registered by a UV-Visible spectrophotometer (Hitachi UV-4100, Hitachi, Tokyo, Japan), in which BaSO_4_ was used as the reflectance standard. Surface composition and chemical states were conducted using X-ray photoelectron spectroscopy (XPS) (Thermo ESCALAB 250XI, Thermo Fisher Scientific, Waltham, MA, USA) equipped with an Mg Kα X-ray source (1253.6 eV) under a vacuum pressure <10^−6^ Pa. Photoluminescence (PL) spectra of the samples were measured by a fluorescence spectrophotometer (FLsp920, British Edinburgh, Edinburgh, UK) at room temperature using Xe lamp as an excitation light source.

Electron spin resonance (ESR) signal of the radicals spin trapped by 5,5-dimethyl-1-pyrroline-*N*-oxide (DMPO, supplied from Sigma Co., Ltd., Saint Louis, MO, USA) was recorded using JES FA200 spectrometer (JEOL Ltd., Tokyo, Japan ). In detail, the sample (5 mg) was dispersed in the solvent benzotrifluoride (BTF, 5 mL). Then, 25 mL DMPO/toluene solution (1:10, *v*/*v*) was added and oscillated to achieve the well-blended suspension. The settings for the ESR spectrometer were as follows: center field = 3507 G, microwave frequency = 9.84 GHz and power = 6.36 mW.

### 2.4. Adsorption Experiments

To investigate the adsorption performance of the prepared photocatalyst, the experiment of adsorption of MO on photocatalyst was performed. 0.01 g of the photocatalyst was added into 50 mL, 30.0 mg/L of MO solution and then stirred the solution at 25 °C for 60 min in the dark. The suspension obtained was filtered, and the concentrations of MO were measured using a UV5100 spectrophotometer at 466 nm. The amount of MO adsorbed (*q_t_*) on samples at the same time intervals was calculated as follows:

(1) $$q_{t}=\frac{(C_{0}-C_{t})V}{m}$$

where *C*_0_ and *C_t_* (mg/L) are the concentrations of MO at initial and the same time intervals, respectively. *V* is the volume of the aqueous solution (L), and *m* (g) is the weight of the added photocatalyst.

### 2.5. Photocatalytic Activity for MO Degradation

Photocatalytic experiments were performed to investigate the degradation of MO solution over photocatalyst by using a 500 W Xe lamp (power adjustment range: 100~1000 W; any irradiation below 420 nm was removed using a cut-off filter) as the visible light source. The power of Xe lamp was kept 500 W and an average irradiation intensity of 350 W/m^2^ was maintained throughout the experiments. The distance between the reactor and lamp housing is 8.5 cm. The temperature was controlled at about 25 °C using cold trap and cooling water circulation pump during the photocatalytic reaction. 0.01 g of photocatalyst was added to 50 mL of MO solution (30 mg/L). The mixture was continuously magnetically stirred and kept in the dark for 60 min to allow adsorption-desorption equilibrium between MO and the photocatalyst. Successively, the suspension was exposed under visible light in order to degrade MO. After degradation experiment, each sample was immediately filtered to remove photocatalyst for analysis. The concentration of MO in clear liquid was measured. The degradation efficiency (η) of MO can be calculated as follows:

(2) $$\eta =\frac{C_{0}-C_{t}}{C_{0}}\times 100\%$$

where *C*_0_ and *C_t_* (mg/L) are the concentrations of MO at the initial and different irradiation times, respectively.

### 2.6. Radicals Scavenging Experiments

To further study the photocatalytic mechanisms of photocatalyst, the radical scavenging experiments were carried out. The holes (h^+^), hydroxyl radical (·OH), the electrons (e^−^) and superoxide radical (·O_2_^−^) are trapped by adding ammonium oxalate (AO) (h^+^ scavenger), tert-butanol (*t*-BuOH) (·OH scavenger), K_2_S_2_O_8_ (e^−^ scavenger) and *p*-benzoquinone (*p*-BQ) (·O_2_^−^ scavenger) into the reaction solution, respectively. Typically, 0.01 g of photocatalyst and 10 mM of radical scavengers were placed into 50 mL of 30 mg/L dye solution, then, the suspension was irradiated under the same conditions of photocatalytic experiments. Finally, the degradation efficiency (*η*) of dye can be calculated to determine the main role of active species.

## 3. Results and Discussion

### 3.1. X-ray Diffraction

**Figure 1:**
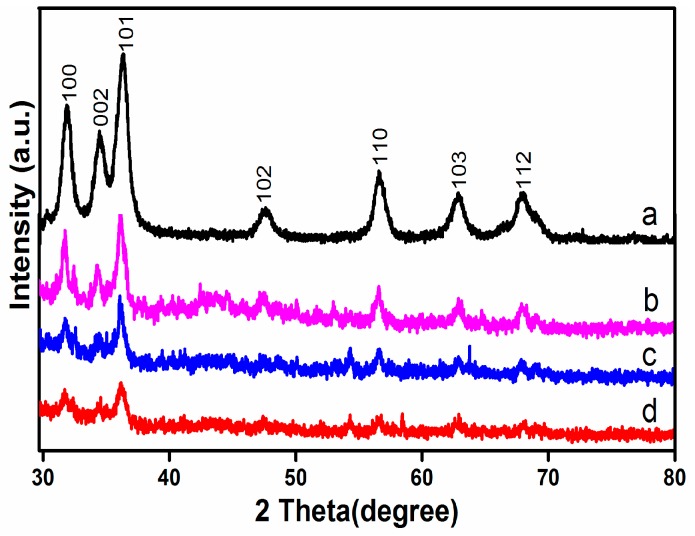
XRD patterns of Ag-N-ZnO (**a**), Ag-N-ZnO/CHAC (**b**), Ag-N-ZnO/CAC (**c**), and Ag-N-ZnO/AAC (**d**).

The XRD patterns of all samples prepared are shown in Figure 1. The peak at 2*θ* = 31.81°, 34.44°, 36.21°, 47.60°, 56.62°, 63.01°, and 67.97° correspond to the (100), (002), (101), (102), (110), (103), and (112) planes of hexagonal wurtzite structure of ZnO. All pertinent diffraction data for ZnO matched well with the reported data (JCPDS 36-1451) [23]. Evidently, the Ag-N-ZnO and Ag-N-ZnO/ACs composites with different ACs loading have the similar XRD patterns, which implied that the addition of Ag, N and AC does not change the phase structure of ZnO. However, no apparent characteristic diffraction peaks of Ag and N were observed in the XRD patterns of all samples prepared, which could be attributed to the low doping amount. Similarly, Bhirud et al. [15] have reported this phenomenon in their studies. Moreover, the characteristic diffraction peak of AC was not detected in the Ag-N-ZnO/AC composites, which is probably due to the relatively low diffraction intensity of AC [24]. Compared with Ag-N-ZnO, the peak intensity of Ag-N-ZnO/AC reduced, which was attribute to the lower amount of crystalline material (ZnO) in a matrix of amorphous matter (AC).

The average crystallite sizes of photocatalysts obtained were calculated from the hexagonal wurtzite (101) peaks using the Debye-Scherrer formula, and the estimated lattice spacing are listed in Table 1. All Ag-N-ZnO/ACs presented smaller crystallite sizes than Ag-N-ZnO because AC acts as barrier to the inhibitive action of crystal growth [22]. The particle size of Ag-N-ZnO/AHAC was 12.1 nm, which was the smallest among all samples. Smaller crystallite size could be conducive to enhancing the photocatalytic activity of photocatalyst. It is similar to the report of Wang et al. [10].

### 3.2. N_2_ Adsorption-Desorption

**Figure 2:**
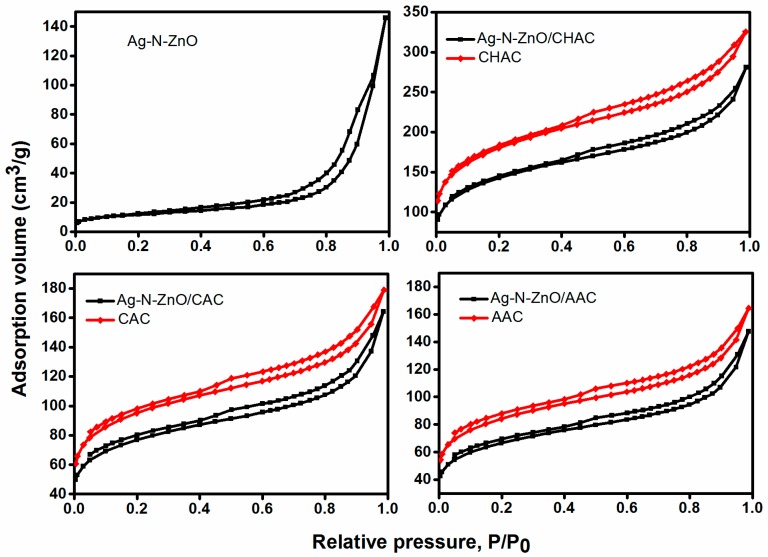
N_2_ adsorption/desorption isotherms curves of Ag-N-ZnO, Ag-N-ZnO/CHAC, Ag-N-ZnO/CAC and Ag-N-ZnO/AAC.

N_2_ adsorption-desorption isotherms and Barret Joyner Halenda (BJH) desorption pore distribution of all samples prepared are shown in Figure 2. In reference to the international union of pure and applied chemistry (IUPAC) classification, the isotherms of Ag-N-ZnO and Ag-N-ZnO/ACs belong to type IV with type H4 hysteresis loops which indicates the existence of mesoporous structure (2–50 nm) The surface area and porous structure of Ag-N-ZnO, Ag-N-ZnO/CHAC, Ag-N-ZnO/CAC, and Ag-N-ZnO/AAC were determined using nitrogen gas adsorption-desorption isotherms. Table 1 shows the parameters of their physical properties such as specific surface area, total pore volume and average pore diameter. As seen from Table 1, the average pore diameter of Ag-N-ZnO and Ag-N-ZnO/ACs were 13.13 nm and 1.35 nm, which further confirmed the presence of mesopores of Ag-N-ZnO and micropores of Ag-N-ZnO/ACs, respectively. The pore channels allow light scattering in the interior and strengthen absorption light [10]. Compared with pure ACs, the specific surface area of all Ag-N-ZnO/ACs decreased, which was the result of two factors: (1) The crystal sizes were larger than the average pore size of AC. Thus, the large size particles mostly deposited on the surface of AC and led to decrease in the surface area of photocatalysts; and (2) the surface area of the composites decreased because ZnO has a markedly lower surface area with respect to ACs. Meanwhile, Ag-N-ZnO/ACs possess higher specific surface area than Ag-N-ZnO, and the BET surface area of Ag-N-ZnO/CHAC is the largest. The larger surface area can help concentrate the MO molecules and the MO molecules approach to the photo-active sites [10,13].

### 3.3. UV-Vis Diffuse Absorption Spectra

**Figure 3:**
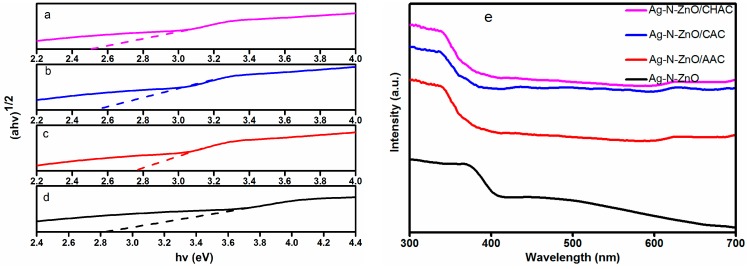
Direct band gap of (**a**) Ag-N-ZnO/CHAC, (**b**) Ag-N-ZnO/CAC, (**c**) Ag-N-ZnO/AAC (**d**) Ag-N-ZnO and (**e**) UV-vis diffuse absorption spectra of all photocatalysts.

The photoabsorption behavior of the synthesized composite photocatalysts was investigated by using UV-vis diffuse absorption spectra. The results are given in Figure 3d. The band gap can be retrieved from Kubelka-Munk elaboration by plotting (*ahν*)^1/2^ versus energy of light (*hν*) as shown in Figure 3a–d, where a is the absorption coefficient. The results are given in Table 1. The band gaps of Ag-N-ZnO, Ag-N-ZnO/CHAC, Ag-N-ZnO/CAC and Ag-N-ZnO/AAC are 2.83, 2.52, 2.55, and 2.77 eV, respectively. This phenomenon could be attributed to the fact that Ag-N-ZnO/CHAC has an abundant pore structure, which allows light to scatter inside [10] and the absorption of CHAC at slightly higher wavelengths than that of pure ZnO. The pure ZnO has strong absorption at a wavelength of 368 nm, indicating the band gap of 3.37 eV cited in the literature. Compared with the pure ZnO, the band edge absorption of prepared composite photocatalysts were red shifted and exhibited extended absorption in the visible-light region (>400 nm), which can be attributed to the charge transfer between the Ag 3d or N 2p state as well as to the ZnO conduction or valance bands. The absorption edge is therefore extended to visible light, leading to the generation of electron-hole pairs, which enhances the photocatalytic properties of the composite photocatalyst [25]. The loading of the Ag-N-ZnO on AC results in a narrowing of the band-gap and the raising of the absorbance in the visible region, which is likely due to the formation of Zn-O-C just like carbon doping ZnO [26].

### 3.4. Photoluminescence

**Figure 4:**
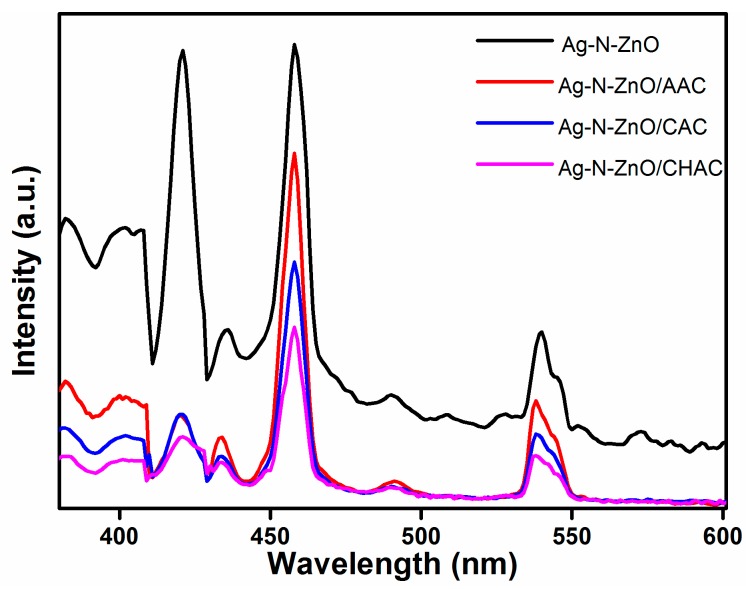
PL spectra of Ag-N-ZnO, Ag-N-ZnO/CHAC, Ag-N-ZnO/CAC and Ag-N-ZnO/AAC.

The photoluminescence (PL) spectra were carried out to estimate the charge recombination and migration efficiency, which is related closely to the photocatalytic properties of a photocatalyst [24]. The PL of all samples is shown in Figure 4. The PL spectra reveals similar curve shapes for all photocatalysts, which indicates that the loading AC affected the intensity of the PL spectra but did not result in a new PL phenomenon. The strong peak at around 410 nm was assigned to the near-band-edge emission of samples [27]. The band at around 435 nm correspond to blue emission, which may originate from some interface traps of radiative defects at the grain boundaries between silver and ZnO grains [28]. All composite materials display similar green light emission peaks centered at 560 nm, which results from the recombination of a singly ionized oxygen vacancy with a photo generated hole [29]. The intensity of the PL spectra decreased in the order of Ag-N-ZnO > Ag-N-ZnO/AAC > Ag-N-ZnO/CAC > Ag-N-ZnO/CHAC. From the XRD analysis, Ag-N-ZnO/CHAC shows the smallest particle size. The smaller particle could facilitate the rapid diffusion of photogenerated carriers to the semiconductor surface, resulting in decreasing the recombination rate of the carrier. A lower PL intensity means a lower electron-hole recombination rate and a higher separation efficiency, hence carriers have a longer lifetime [26]. Therefore, the efficient charge separation and inhibited electron-hole recombination could probably enhance the photocatalytic activity of photocatalysts.

### 3.5. X-ray Photoelectron Spectroscopy

**Figure 5:**
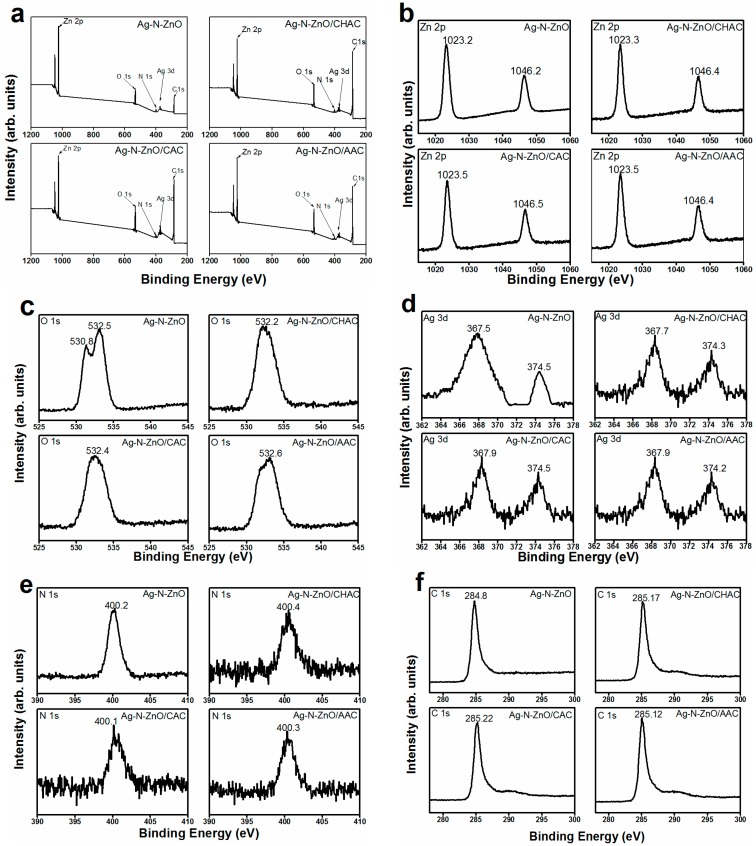
XPS spectra of survey spectra (**a**) Zn 2p (**b**), O 1s (**c**), Ag 3d (**d**), N 1s (**e**) and C 1s (**f**) levels of Ag-N-ZnO, Ag-N-ZnO/CHAC, Ag-N-ZnO/CAC and Ag-N-ZnO/AAC.

To investigate the surface chemical composite and chemical state of all samples, the X-ray Photoelectron Spectroscopy (XPS) of photocatalysts was studied (Figure 5). The binding energies were calibrated by using C 1s (285.77 eV) as the reference. The typical XPS survey of all products shows that the composite photocatalyst only consists of Zn, O, Ag, N, and C. No other elements were detected. The carbon peak of Ag-N-ZnO (C 1s = 284.8 eV) was attributed to hydrocarbons from the XPS instrument. Nevertheless, the high-resolution XPS of Zn 2p, O 1s, and Ag 3d, C 1s shows that the binding energies of these electrons in the case of Ag-N-ZnO and Ag-N-ZnO/ACs were slightly different, due to a strong interaction between the Ag, N, and ZnO nanoparticles and different ACs as carrier. In the Zn 2p XPS spectrum, the two specific peaks located at around 1023.3 eV for 2p_3/2_ and 1046.4 eV for 2p_1/2_, respectively. This outcome indicates that zinc element existed mainly as state of Zn^2+^ state in the all samples [30]. The O 1s XPS spectra of different photocatalysts were highly similar. The peaks at approximately 530.8 eV of the samples were attributed to lattice oxygen present in ZnO, whereas the main peak at around 532.0 eV was assigned to hydroxyl groups absorbed onto the surface of the photocatalyst, which are related to the formation of hydroxyl radicals [31]. The presence of surface hydroxyl groups is in favor of the trapping of photogenerated electrons and holes to produce ·OH, resulting in enhanced photocatalytic degradation. The core level Ag 3d spectrum of Ag in composite photocatalyst consisted of two peaks at 367.5 and 374.5 eV corresponding to 3d_5/2_ and 3d_3/2_ respectively, which can contribute to metallic silver (Ag^0^) in the photocatalyst [32,33]. Effective nitrogen doping onto ZnO crystals can be confirmed by the obvious XPS signal of N 1s. The peaks at around 400.3 eV may be attributed to -Zn-N-O- or -O-Zn-N- [30,34], indicating that some O atoms were substituted by nitrogen. These results strongly show the successful doping of nitrogen in ZnO crystals. Furthermore, the similar peaks indicated that the incorporation of Ag did not induce an obvious change in the chemical state of N. The C 1s peaks of Ag-N-ZnO/CHAC, Ag-N-ZnO/CAC, and Ag-N-ZnO/AAC located at around 285.2 eV was signed to the sp^2^-hybridized carbon, which is attributed to the AC present in the synthesized composite material [10].

### 3.6. Adsorption Studies

**Figure 6:**
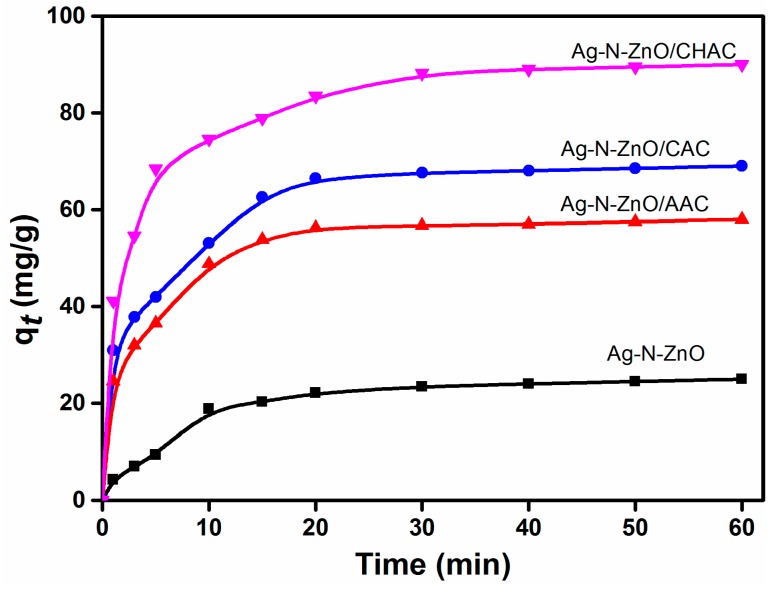
Adsorption kinetics of MO on the four composite photocatalysts.

The adsorption kinetic curves of four composite photocatalysts were researched (Figure 6). It can be found that the four photocatalysts have similar adsorption behaviors. The adsorption process undergoes two steps: MO is rapidly adsorbed onto the photocatalyst surface within the first 10 min period. At the second step, the adsorption rate becomes slower until the adsorption equilibrium is reached because the number of available active sites gradually decreased. The adsorption process can almost reach equilibrium in about 60 min. Among these photocatalysts, Ag-N-ZnO/CHAC composite photocatalyst exhibits the highest amounts of adsorption due to its large specific surface area [10].

Additionally, pseudo-first-order and pseudo-second-order models were introduced to study the adsorption kinetics of MO onto photocatalysts, whereas the intra-particle diffusion model was employed to further analyze the adsorption kinetic results and to verify whether the intra-particle diffusion was the only rate-determining step.

Pseudo-first-order model:

(3) $$q_{t}=q_{e}\left(1-e^{-k_{1}t}\right)$$

Pseudo-second-order model:

(4) $$q_{t}=\frac{k_{2}q_{e}^{2}t}{1+k_{2}q_{e}t}$$

Intra-particle diffusion model:

(5) $$q_{t}=k_{id}t^{1/2}+C$$

where *q_e_* (mg/g) and *q_t_* (mg/g) are the amounts of MO adsorbed at equilibrium and at time *t* (min), respectively; *k*_1_ (min^−1^), *k*_2_ (g·mg^−1^·min^−1^) and *k_id_* (mg·g^−1^·min^−1/2^) are the rate constants of the pseudo-first-order, pseudo-second-order and intra-particle diffusion models of the adsorption process, respectively. *C* is the intercept of the line, which is proportional to the boundary layer thickness.

Table 2 shows the parameters of the three kinetic models. The adsorption kinetics of MO onto composite photocatalysts can be better described using the pseudo-second-order model than the pseudo-first-order model. This phenomenon was due to the higher correlation coefficient values. Furthermore, the obtained theoretical values (*q_e_*,_cal_) calculated from the pseudo-second-order model matched well with the experimental data (*q_e_*,_exp_). In addition, based on intra-particle diffusion model, the adsorption process consists of two steps. The first stage was related to the boundary layer diffusion of MO from the bulk solution to the external surface of the photocatalyst. The second stage was corresponded to the gradual diffusion of Mo through the boundary layer into the internal pores of photocatalyst until the adsorption equilibrium was reached. Similarly, Hou et al. have reported this phenomenon in their studies [35]. The lines did not pass through the origin, indicating that the intra-particle diffusion is not the exclusive rate-determining step [36].

Considering the influence of adsorption on photocatalysis, we put the catalysts into the MO solution in darkness before the photocatalytic runs to allow the adsorption of MO on the catalysts at equilibrium conditions. According to adsorption experiments, the adsorption process can almost reach equilibrium in about 60 min, hence the mixture was kept in the dark for 60 min.

### 3.7. Photocatalytic Activity

**Figure 7:**
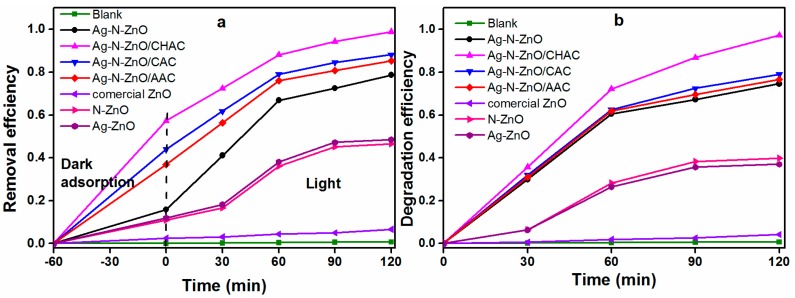

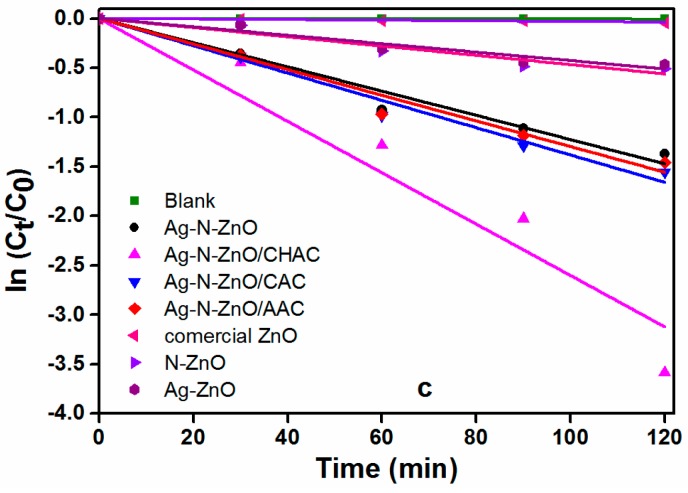
Photocatalytic degradation efficiency (**a**) (30 mg/L initial concentration of MO solution), photocatalytic degradation efficiency of dark adsorption eliminated (**b**) (initial concentration of MO solution after dark adsorption experiment), and kinetic curves (**c**) of photocatalytic degradation of MO over different photocatalysts under visible light irradiation.

The photocatalytic performance of the different prepared photocatalysts was estimated by the degradation of MO under visible light irradiation after dark adsorption for 60 min (Figure 7a). A blank experiment was carried out under visible light irradiation for 120 min in the absence of photocatalysts, which indicated that the direct photolysis of MO could be negligible under visible light irradiation. The commercial ZnO exhibits virtually no activity under visible light irradiation. Compared with commercial ZnO, the photocatalytic activity of Ag-ZnO and N-ZnO were enhanced. This phenomenon is due to the fact that Ag can act as an electron scavenger to trap the photogenerated electrons, thereby decreasing the recombination rate of electron-hole pairs [24]. Moreover, N doping can provide an impurity energy level above the valence band of ZnO, thereby responding to the visible light and reducing the band gap [37]. The photocatalytic activity of Ag-N-ZnO is higher than single-doped ZnO, which is attributed to intense visible light absorbance and a lower recombination rate of electrons and holes [38]. The Ag-N-ZnO loaded on AC exhibited a better photocatalytic degradation efficiency compared with pure Ag-N-ZnO. This result is reasonable on the basis of the two following factors: (1) The smaller particle size has more active sites at a constant amount of photocatalyst, which leads to an increase in the adsorption of O_2_ and –OH, hence the generation of radicals is increased [23] and (2) AC may contribute to MO molecules gathered around Ag-N-ZnO particles in low concentration solution of MO [10]. These factors resulted in high photocatalytic activity. Among the all composite photocatalysts, the Ag-N-ZnO/CHAC exhibits the highest photocatalytic activity and the degradation efficiency of MO reached 98.82% within 120 min under visible light radiation. Moreover, when the adsorption of the photocatalyst was eliminated, the degradation efficiency of MO can still reach 97.23% within 120 min under visible light radiation (Figure 7b). This is due to three reasons: (1) The particle size of Ag-N-ZnO/CHAC is the smallest, which is in favor of adsorption of O_2_ and –OH to generate ·OH and ·O_2_^−^ [23]; (2) The surface area of Ag-N-ZnO/CHAC is the largest, which may contribute to more MO molecules gathering around Ag-N-ZnO [10]; and (3) The intensity of the PL of Ag-N-ZnO/CHAC is the weakest, which indicates a decrease in the recombination rate of electrons and holes, thus increasing the life of electrons and holes [26]. These factors resulted in a highest photocatalytic activity of Ag-N-ZnO/CHAC.

Furthermore, the photodegradation kinetics of MO on the four composite photocatalysts were investigated (Figure 7c). The kinetic curves of various photocatalysts follow First order kinetics and the degradation rate constants of all samples are presented in Table 3. The apparent rate constant of photocatalyst is in the following order: Ag-N-ZnO/ACs > Ag-N-ZnO > N, or Ag single-doped ZnO > commercial ZnO, which indicated that N, and Ag co-doped ZnO supported on AC can enhance the photacatalytic activity of the photocatalyst. Ag-N-ZnO/CHAC achieved excellent photocatalytic performance with a *k* value of 0.0260 min^−1^, which was 2.11 times that of Ag-N-ZnO (*k* = 0.0123 min^−1^).

### 3.8. Photogenerated Active Radical Species

**Figure 8:**
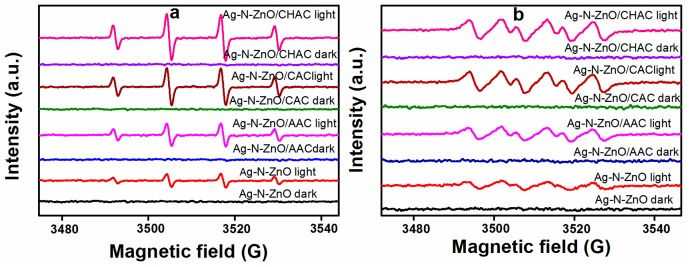
ESR spectra of radical adducts trapped by DMPO over the different photocatalyst dispersions under visible light irradiation; (**a**) DMPO-·OH formed in irradiated aqueous dispersions; (**b**) DMPO-O_2_**^•^**^−^ formed in irradiated methanol dispersions.

In order to detect the involvement of radical species generated in the photocatalytic process, the ESR spin-trap with DMPO technique has also been performed on illuminated photocatalysts [39]. The results are displayed in Figure 8. Dark treatment experiments show that no signals (·OH and ·O_2_^−^) were detected in the presence of photocatalysts. As shown in Figure 8a, under visible light irradiation, the signals of DMPO-·OH for all samples can be clearly observed, and the intensity increases with the addition of AC. This finding is further defined by the formation of ·OH during the process of photocatalytic degradation of dyes in the presence of photocatalysts and visible light irradiation. Moreover, the superoxide radical (·O_2_^−^) was observed, as displayed in Figure 8b. When photocatalysts were irradiated with visible light, Ag-N-ZnO/CHAC could generate stronger and more obvious peaks of the DMPO-·O_2_^−^ species than other samples, indicating that Ag-N-ZnO/CHAC can generate more ·O_2_^−^ radicals. In particular, under identical operational conditions, the Ag-N-ZnO/CHAC composite photocatalyst exhibits the strongest intensities of ·OH and ·O_2_^−^, which seems consistent with the more efficient charge separation and transfer from Ag-N-ZnO/CHAC as investigated by the above photoluminescence (PL) spectrum analysis. The smaller particle size of Ag-N-ZnO/CHAC could facilitate the rapid diffusion of photogenerated carriers to the semiconductor surface, resulting in a decrease of the recombination rate of the carrier. Therefore, more electrons and holes can react with O_2_ and –OH to form ·OH and ·O_2_^−^, respectively, resulting in stronger intensities of ·OH and ·O_2_^−^. This result is in strong agreement with photocatalytic activity.

### 3.9. Mechanism of Photodegradation

**Figure 9:**
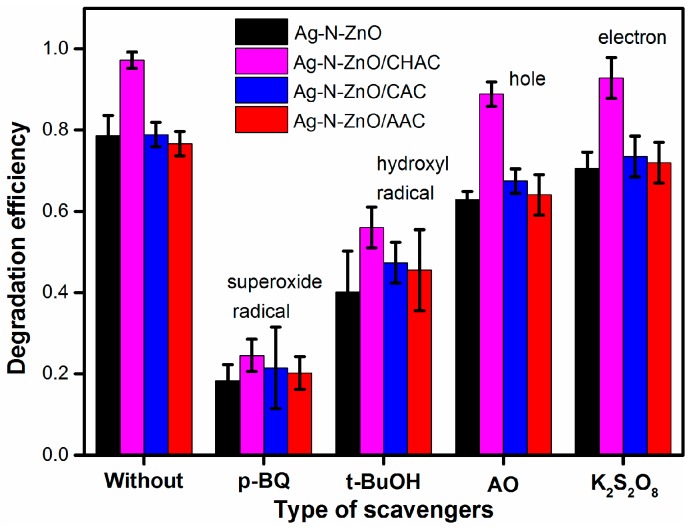
The removal rate of MO over Ag-N-ZnO/ACs in the presence of various scavengers.

To further understand the underlying mechanism involved in the degradation of MO on the photocatalysts under visible light irradiation, we conducted radical, hole, and electron scavenging experiments to detect the major role of reactive species in the photocatalytic process. Tert-butanol (t-BuOH) was adopted to quench the hydroxyl radical (·OH) [24], p-benzoquinone (p-BQ) was used as the superoxide radical (·O_2_^−^) scavengers [10], ammonium oxalate (AO) was introduced as the scavenger of holes (h^+^) [23], and the electrons (e^−^) were trapped by K_2_S_2_O_8_ [39]. The degradation rate of MO over photocatalysts in the presence of the scavengers is presented in Figure 9. The addition of t-BuOH and p-BQ markedly decreased the removal rate of MO. By contrast, the removal efficiency of MO only slightly changed in the photocatalytic process in the presence of AO and K_2_S_2_O_8_. The removal rate of the MO over photocatalysts in the presence of scavengers followed the order: p-BQ < t-BuOH< AO < K_2_S_2_O_8_. Therefore, the reactive species ·OH and ·O_2_^−^ play more important roles in the photocatalytic degradation process of MO over composite photocatalysts, which agrees with the common viewpoint on the photocatalytic degradation of azo dyes on photocatalysts [40,41].

**Figure 10:**
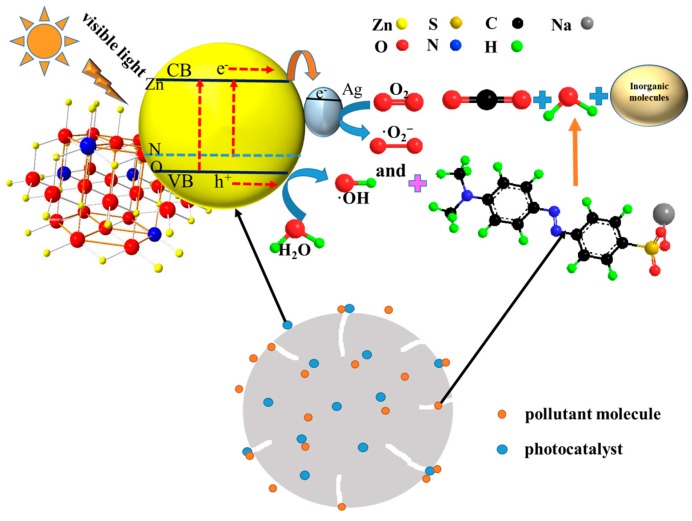
Mechanism for photocatalytic degradationaction of MO on the Ag-N-ZnO/AC photocatalyst.

The enhanced photocatalytic activity of the Ag-N-ZnO/AC composite photocatalyst can be understood as follows and the possible mechanisms are illustrated in Figure 10. As the photocatalyst is irradiated by visible light, ZnO is activated to produce the photogenerated electrons and holes. Then the photogenerated electrons and holes react with O_2_ and -OH, respectively, to form ·OH and ·O_2_^−^. The MO is then decomposed by ·OH and ·O_2_^−^. The high photocatalytic activity of the Ag-N-ZnO/AC photocatalysts under visible light may be attributed to N and Ag dopants as well as to the AC. Firstly, this phenomenon is achieved by substituting O sites with N, which can form impure energy levels. Thus, electrons transfer from the valence band (VB) to the N 2p and from the N 2p to the conduction band (CB), inducing a longer wavelength of the absorbed photon [13]. Secondly, the photogenerated electrons excited by visible light were trapped by Ag after they were transferred to the conduction band of the prepared composites, which prevented their recombination with holes [35]. Thirdly, AC acts as a barrier to the inhibitive action of ZnO crystal growth, which increased the surface of contact between MO and the photocatalyst. AC could absorb a large part of irradiation, which traded off its advantage as an adsorptive support to concentrate MO molecules. Some hot-spots could be formed on the surface of the AC particles, and the MO molecules around the “hot spots” could be decomposed in the presence of O_2_ dissolved in water [24]. These factors all resulted in the high photocatalytic activity of the Ag-N-ZnO/AC composite photocatalyst.

### 3.10. Stability of Composite Photocatalyst

**Figure 11:**
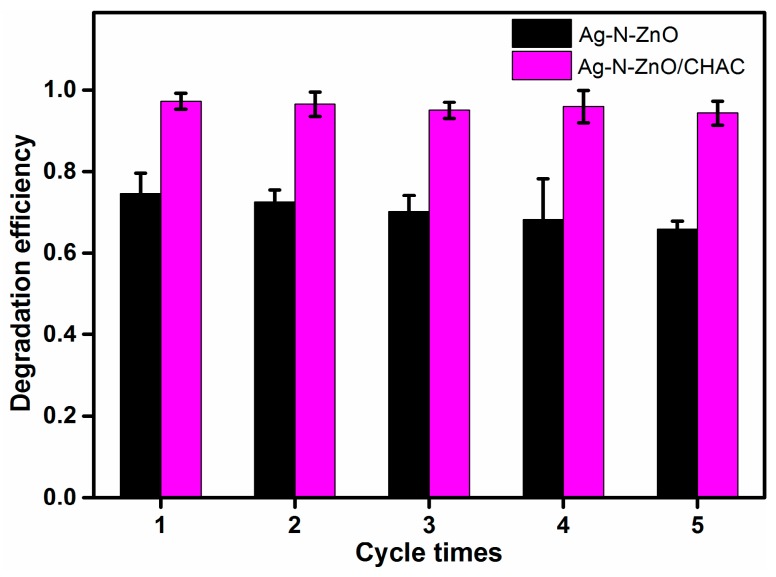
Recycled photoactivity testing of Ag-N-ZnO and Ag-N-ZnO/CHAC photocatalysts under visible light irradiation.

In addition to photocatalytic properties, the stability and recyclability of the photocatalyst are important for its potential application. To evaluate the stability of the photocatalyst, the photocatalytic degradation of recycled photocatalysts was carried out under visible light irradiation. The photocatalyst was collected after the photocatalytic reaction, then washed with distilled water and ethanol and dried in an oven at 80 °C before reuse. As shown in Figure 11, theAg-N-ZnO/CHACphotocatalyst retained its high photocatalytic activity, and still the degradation efficiency of MO reached 94.32% after five recycle runs. The photocatalytic activity of Ag-N-ZnO/CHAC only minimally decreased, which could be due to three reasons: (1) The pore of the composite photocatalyst were blocked resulting in the decrease of the ability of photocatalyst to concentrate MO molecules; (2) The photocatalyst was lost during the cycle processes; and (3) The partial active sites were deactivated or blocked due to not complete degradation of MO and MO intermediates during each previous cycle. The degradation efficiency of MO over Ag-N-ZnO decreased from 74.6% to 65.88% after five recycle runs. This phenomenon is mainly attributed to a higher loss of Ag-N-ZnO photocatalysts during the difficult separation processes. These results indicate that AC as supported materials can not only improve photocatalytic activity, but also improve the stability of the photocatalyst.

### 3.11. Correlation Analysis

**Figure 12:**
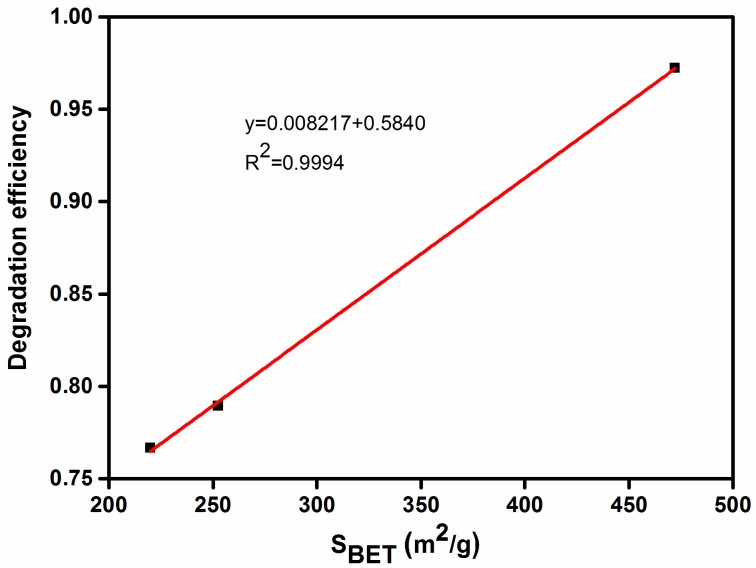
Correlation curve between BET surface area of Ag-N-ZnO/CHAC, Ag-N-ZnO/CAC and Ag-N-ZnO/AAC and remove efficiency of MO.

It is important to study the correlation between the physical and chemical properties of the photocatalyst and its degradation properties. Figure 12 shows that the S_BET_ of Ag-N-ZnO/ACs presented a good positive correlation with MO removal rate (*R*^2^ = 0.9994). A large specific surface area of the photocatalyst will result in a high removal rate of MO. This result is consistent with those obtained above. Therefore, the best photocatalytic degradation of MO was observed over Ag-N-ZnO/CHAC.

## 4. Conclusions

All composite photocatalysts were successfully prepared through sol-gel and ultrasonic method. Ag-N-ZnO/ACs exhibited higher photocatalytic activity for MO than for the unsupported Ag-N-ZnO. Among all Ag-N-ZnO/ACs, Ag-N-ZnO/CHAC exhibited the highest photocatalytic activity for MO. The highest surface area and the strongest adsorption performance of CHAC resulted in inhibiting the growth of ZnO crystallite. The smaller particle size of Ag-N-ZnO/CHAC could facilitate the rapid diffusion of photogenerated carriers to the semiconductor surface, resulting in decreasing the recombination rate of the carrier. In addition, the highest photocatalytic activity of Ag-N-ZnO/CHAC is due to the fact that Ag-N-ZnO/CHAC has an abundant pore structure and CHAC absorbs at slightly higher wavelengths than that of ZnO. The adsorption of MO over photocatalyst reached equilibrium in about 60 min and fitted the pseudo-second-order kinetics. The degradation of MO on photocatalyst followed first order kinetics. Reactive species ·OH and ·O_2_^−^ played more important roles in the photocatalytic degradation process of MO over the composite photocatalysts. The Ag-N-ZnO/CHAC photocatalyst retained higher photocatalytic activity than the unsupported Ag-N-ZnO after five recycle runs. Hence, the Ag-N-ZnO/CHAC photocatalyst could be considered a promising material for application in the removal of MO from aqueous solution.

## Figures and Tables

**Table 1:** Characteristics of different photocatalysts.

Samples	Crystallite Size (nm) ^a^	D (101) (nm) ^b^	*S*_BET_ (m^2^/g)	*V*_t_ (cm^3^/g)	Average Pore Size (nm)	Band Energy (eV)
Ag-N-ZnO	18	0.2477	40	0.2258	13.13	2.83
Ag-N-ZnO/CHAC	12	0.2480	472	0.4348	1.35	2.52
Ag-N-ZnO/CAC	14	0.2482	252	0.2542	1.35	2.55
Ag-N-ZnO/AAC	17	0.2484	219	0.2284	1.35	2.77
CHAC	-	-	593	0.5037	1.35	-
CAC	-	-	312	0.2769	1.35	-
AAC	-	-	276	0.2545	1.35	-

*S*_BET_, specific surface area obtained by BET equation; *V*_t_ total pore volume; Average pore size from pore size distribution determined by Barret Joyner Halenda (BJH) method. ^a^ Based on XRD analysis, using the Scherrer equation: crystalline size = 0.89*λ*/*β*cos*θ*, where *λ* = 0.154 nm, *β* is the full width at half maximum (FWHM), and *θ* is the diffraction angle. ^b^ Based on XRD analysis using Bragg’s law: lattice spacing (D) = *λ*/(2 × sin*θ*). Band energy retrieved from Kubelka-Munk elaboration by plotting (*ahν*)^1/2^ versus energy of light (*hν*).

**Table 2:** Kinetic parameters of MO adsorption onto the four composite photocatalysts.

Samples	*q_e_*_,exp_ (mg/g)	Pseudo-First-Order			Pseudo-Second-Order			Intra-Particle Diffusion Model					
		*q_e_*_,cal_ (mg/g)	*k*_l_ (min^−1^)	*R*^2^	*q_e_*_,cal_ (mg/g)	*k*_2_ (g mg^−1^ · min^−1^)	*R*^2^	*k_i_*_,1_ (mg g^−1^ · min^−1/2^)	*C*_1_ (mg/g)	*R*^2^	*k*_i,2_ (mg g^−1^ · min^−1/2^)	*C*_2_ (mg/g)	*R*^2^
Ag-N-ZnO	25.14 ± 0.02	28.31 ± 0.03	0.1200	0.9827	24.53 ± 0.05	0.0054	0.9887	4.1580	0.0140	0.9978	1.2973	15.5364	0.9086
Ag-N-ZnO/CHAC	90.42 ± 0.04	84.70 ± 0.02	0.3964	0.9413	90.71 ± 0.02	0.0073	0.9862	29.9700	3.8097	0.9566	3.3120	66.7464	0.8387
Ag-N-ZnO/CAC	69.17 ± 0.02	66.10 ± 0.04	0.3664	0.9117	71.24 ± 0.02	0.0068	0.9602	18.6600	4.5380	0.8610	2.7166	50.2340	0.5983
Ag-N-ZnO/AAC	58.28 ± 0.04	56.05 ± 0.04	0.2734	0.9447	60.39 ± 0.04	0.0070	0.9764	16.2880	3.0551	0.9140	1.5769	46.8706	0.6427

**Table 3:** First order kinetic constants and relative coefficients for photocatalytic degradation of MO over the samples.

Samples	*k* (min^−1^)	*R*^2^
Blank	6.24 ± 0.12 × 10^− 5 a^	0.9945
Commercial ZnO	3.28 ± 0.10 × 10^− 4 a^	0.9834
Ag-ZnO	4.28 ± 0.13 × 10^− 3 b^	0.9697
N-ZnO	4.69 ± 0.08 × 10^− 3 b^	0.9698
Ag-N-ZnO	1.23 ± 0.04 × 10^− 2 c^	0.9854
Ag-N-ZnO/CHAC	2.60 ± 0.05 × 10^− 2 d^	0.9661
Ag-N-ZnO/CAC	1.38 ± 0.03 × 10^− 2 c^	0.9912
Ag-N-ZnO/AAC	1.30 ± 0.02 × 10^− 2 c^	0.9874

Different lowercase letters show significance between samples and *k* at *p* < 0.05 using least significant difference (LSD).
